# LPIN1 Induces Gefitinib Resistance in EGFR Inhibitor-Resistant Non-Small Cell Lung Cancer Cells

**DOI:** 10.3390/cancers14092222

**Published:** 2022-04-29

**Authors:** Jung Hee Cho, Yeon-Mi You, Han Koo, Dong Chul Lee, Young Il Yeom, Kyung Chan Park

**Affiliations:** 1 Personalized Genomic Medicine Research Center, Korea Research Institute of Bioscience & Biotechnology (KRIBB), Daejeon 34141, Korea; junghee.cho@net-targets.com (J.H.C.); uyeonmi@kribb.re.kr (Y.-M.Y.); koohan123@naver.com (H.K.); dclee@kribb.re.kr (D.C.L.); 2 Department of Functional Genomics, University of Science and Technology, Daejeon 34113, Korea

**Keywords:** LPIN1, tyrosine kinase inhibitors, gefitinib, drug resistance, non-small cell lung cancer

## Abstract

**Simple Summary:**

Here, we attempted to identify targets that could be used to overcome resistance toward epidermal growth factor receptor (EGFR) tyrosine kinase inhibitors in non-small cell lung cancer (NSCLC). To accomplish this, we chose LPIN1 among the candidate targets that were identified from a previously performed genome-wide RNAi screening assay and validated it as a key factor regulating gefitinib resistance in EGFR-mutant NSCLC cells. We confirmed that LPIN1 depletion increased gefitinib sensitivity in drug-resistant H1650 NSCLC cells, as well as patient-derived YL05 lung cancer cells. Moreover, we found that LPIN1 expression was induced following gefitinib treatment, and activities of protein kinase C delta and nuclear factor kappa B, and lipid droplet formation were induced in an LPIN1-dependent manner. Additionally, we validated that targeting LPIN1 synergistically retarded tumor growth in an in vivo mouse xenograft model.

**Abstract:**

Drug resistance limits the efficacy of targeted therapies, including tyrosine kinase inhibitors (TKIs); however, a substantial portion of the drug resistance mechanisms remains unexplained. In this study, we identified LPIN1 as a key factor that regulates gefitinib resistance in epidermal growth factor receptor (EGFR)-mutant non-small cell lung cancer (NSCLC) cells. Unlike TKI-sensitive HCC827 cells, gefitinib treatment induced LPIN1 expression and increased diacylglycerol concentration in TKI-resistant H1650 cells, followed by the activation of protein kinase C delta and nuclear factor kappa B (NF-κB) in an LPIN1-dependent manner, resulting in cancer cell survival. Additionally, LPIN1 increased the production of lipid droplets, which play an important role in TKI drug resistance. All results were recapitulated in a patient-derived EGFR-mutant NSCLC cell line. In in vivo tumorigenesis assay, we identified that both shRNA-mediated depletion and pharmaceutical inhibition of LPIN1 clearly reduced tumor growth and confirmed that gefitinib treatment induced LPIN1 expression and LPIN1-dependent NF-κB activation (an increase in p-IκBα level) in tumor tissues. These results suggest an effective strategy of co-treating TKIs and LPIN1 inhibitors to prevent TKI resistance in NSCLC patients.

## 1. Introduction

Lung cancer, a leading cause of cancer-induced death worldwide, is histologically classified into two main subgroups: small cell lung carcinoma (SCLC) and non-small cell lung carcinoma (NSCLC) [1,2]. Contrary to SCLC patients with rare mutations in epidermal growth factor receptor (EGFR), about 10~30% of NSCLC patients may harbor “activating mutations” in EGFR that causes constitutive activation of the EGFR pathway and provides the benefit of abnormal tumor growth [3,4]. EGFR inhibitors have been developed for targeted therapies to treat NSCLC and are most effective in patients with tumors that are highly dependent on EGFR signaling. However, only 5% of the patients achieve dramatic initial responses (>90% tumor reduction) to treatment, and moreover, most of them eventually become resistant to EGFR inhibitors [4,5,6]. The remaining patients, comprising the majority of patients with NSCLC, respond partially or in a very limited manner to TKI treatment, despite harboring activating EGFR mutations. The development of drug resistance often limits durable clinical responses to therapy. Several mechanisms of innate and acquired resistance have been discovered, including EGFR T790M mutation, MET proto-oncogene amplification, phosphatase and tensin homolog (PTEN) deletion, and a second mutation in the downstream pathway of EGFR [6,7,8,9]. However, it is still crucial to identify the mechanisms of underlying resistance to EGFR-TKIs, particularly in patients with innate resistance.

Highly proliferating cancer cells are often exposed to metabolic stress under limited availability of oxygen and nutrients, which leads to alterations in cellular metabolism for cell growth and survival [10,11,12]. Together with alteration in glucose and glutamine metabolism, lipid metabolism is often dysregulated in cancer. Lipid metabolic reprogramming is an emerging mechanism in cancer cells for membrane biogenesis, energy production, cell survival, and resistance to anti-cancer drugs [13,14,15,16]. Cancer cells undergo de novo lipid synthesis (lipogenesis), resulting in the accumulation of neutral lipids such as triglyceride (TG) stored in lipid droplets (LDs) [14,17]. Recent studies have revealed an association between the LD content and drug resistance [18]. Gefitinib-resistant cell lines express LDs more than TKI-sensitive cell lines do [19]. Increased LD accumulation and fatty acid metabolism are associated with drug sensitivity in breast cancer cells [20]. LDs suppress endoplasmic reticulum stress and apoptosis, which are essential for treatment of lung cancer [18,21]. The protein kinase C (PKC) family, a group of serine-threonine kinases, is intimately associated with lipid metabolism [22,23]. Diacylglycerol (DAG), a precursor of TG synthesis, commonly activates conventional and novel PKC subfamily members, which are involved in a variety of cellular functions, including survival and motility [24,25]. Inhibition of PKC expression or activity results in the inhibition of cancer cell proliferation or cell death [26,27,28]. The EGFR-Y1173-phospholipase C gamma (PLCγ)-DAG axis induces the activation of PKCδ, which is related to cancer cell proliferation, and a potent therapeutic target in EGFR-mutant NSCLC cells [29]. However, the mechanism of the differential response of lipid metabolic reprogramming to drugs in drug-sensitive and drug-resistant cancer cells is not well understood.

LPIN1 is a mammalian Mg^2+^-dependent phosphatidic acid phosphatase (PAP) enzyme that converts phosphatidic acid (PA) to DAG, a precursor of triacylglycerol and phospholipid [30]. Nuclear LPIN1 blocks nuclear translocation of sterol regulatory element-binding protein 1 (SREBP1), thereby inhibiting the expression of lipid synthesis genes [31]. In addition, LPIN1 acts as a transcriptional coactivator of peroxisome proliferator-activated receptor alpha (PPARα) and PPARγ coactivator-1 alpha (PGC-1α), increasing fatty acid oxidation capacity in the liver [32,33]. However, during mTORC1 activation, LPIN1 is phosphorylated, and its nuclear localization is blocked, resulting in the DAG production and promotion of lipid synthesis through nuclear SREBPs [31]. LPIN1 plays a critical role in cancer progression, and its expression is increased in various cancer cells [34,35,36], indicating its role in cell proliferation and tumor growth. However, the underlying cellular mechanisms of LPIN1 in EGFR-TKI drug resistance remain unclear.

Previously, we found LPIN1 as a factor closely related to gefitinib resistance in EGFR-activating mutant NSCLC cells [37]. Here, we identified that LPIN1 contributed to a gefitinib-dependent increase in DAG production and induced resistance signals by activating the PKCδ–NF-κB pathway. We suggest that LPIN1 is a potential target to attenuate resistance towards gefitinib in EGFR-mutant NSCLC and that co-targeting PLCs and LPINs, which takes charge of cellular DAG production, might be an effective way to overcome TKI resistance.

## 2. Materials and Methods

### 2.1. Chemicals and Cell Culture

The following chemicals were suspended in dimethyl sulfoxide: gefitinib (Cayman Chemical, Ann Arbor, MI, USA); LPIN inhibitor propranolol (Selleckchem, Houston, TX, USA); PKCα, PKCβI, PKCβII, and PKCγ inhibitor GF109203X (S7208) (Selleckchem); PKCα, PKCβ, PKCγ, and PKCδ inhibitor Go 6983 (S2911) (Selleckchem); phospholipase C (PLC) inhibitor U73122 (Selleckchem); and etoposide (Sigma-Aldrich, Saint Louis, MO, USA).

Detailed information on the cell lines used is described in our previous publication [37]. HCC827, NCI-H1650, PC-9 and YL05 were maintained in Roswell Park Memorial Institute 1640 medium (RPMI, Welgene, Gyeongsan-si, Republic of Korea, #LM011-01), and HEK293T was cultured in Dulbecco’s modified Eagle’s medium (DMEM, Welgene, #LM001-05) supplemented with 10% fetal bovine serum (Thermo Fisher Scientific Inc, Grand Island, NY, USA) and 1% penicillin-streptomycin (Thermo Fisher Scientific Inc, Waltham, MA, USA). Cells were maintained in an incubator with 5% CO_2_ at 37 °C.

### 2.2. Plasmids, Small Interfering RNA, and Transfection of Nucleic Acids

The psPAX2, pMD2.G (Addgene, Waltertown, MA, USA, #12260, #12259) and a pool of shRNAs targeting LPIN1 or a non-targeting pLKO (Sigma-Aldrich) (Supplementary Table S1) were used for packaging of lentiviral particles. The AccuTarget™ Genome-wide Predesigned siRNAs targeting LPIN1, PKCα, PKCδ, PKCζ, RICTOR and Negative Control siRNA (Bioneer Inc., Daejeon, Korea) (Supplementary Table S1) were used for transient gene-silencing. The transfections of plasmids or two-siRNA mixtures for each gene were performed with Fugene^®^ HD transfection reagent (Promega, Madison, WI, USA) or Lipofectamine^®^ RNAiMAX reagent (Thermo Fisher Scientific Inc., Carlsbad, CA, USA), respectively.

### 2.3. Cell Viability Assay and Colony Forming Assay

Detailed methods are described in the previous publication [37]. Briefly, for cell viability assay, cells on a 96-well plate were treated with vehicle or inhibitors for 5 days. Then, cells were incubated in MTT solution for 1 h in a CO_2_ incubator. After removing the solution, formazan crystals were dissolved in DMSO, and the absorbance was measured at 570 nm using a Luminoskan microplate reader (Thermo Scientific, San Diego, CA, USA). Cell viability was calculated as a percentage value compared to the absorbance of untreated control cells. Data were obtained from three independent experiments, with each performed in triplicate. For the colony forming assay, cells plated onto a 6-well plate were treated with gefitinib or DMSO for 10 days and then stained with 0.5% crystal violet in 20% methanol for 20 min. Images were captured under a microscope using a DP Controller software (Olympus, Tokyo, Japan, version 2.1).

### 2.4. Anchorage-Independent Soft-Agar Assay

For the base agar, 0.6% agar in complete medium was added to a 6-well plate and solidified. For the top agar, 0.3% agar in complete medium containing cells was added on top of the base agar. The cells plated in agar were incubated at 37 °C in a 5% CO_2_ incubator for 2 weeks and treated with medium containing DMSO or gefitinib every 3 days. Colony images were captured under a microscope using a DP Controller software (Olympus, version 2.1).

### 2.5. Western Blot Analysis

The total proteins were obtained by lysing cells in ice-cold radioimmunoprecipitation assay (RIPA) buffer for 30 min on ice. After centrifugation at 10,000 rpm at 4 °C for 15 min, proteins in the supernatant were subjected to 10% or 8% sodium dodecyl sulfate-polyacrylamide gels and then transferred to nitrocellulose membranes. The membranes containing proteins were incubated at 4 °C for 16 h with primary antibodies against p-EGFR (1:1000, Cell Signaling Technology, Danvers, MA, USA, #2236), EGFR (1:1000, Millipore, #06-847), LPIN1 (1:1000, Cell Signaling Technology, #5195), PARP (1:1000, Cell Signaling Technology, #9542), caspase-3 (1:1000, Cell Signaling Technology, #9662), cleaved caspase-3 (1:1000, Cell Signaling Technology, #9664), p-IkBα (1:1000, Cell Signaling Technology, #2859), IkBα (1:1000, Cell Signaling Technology, #4812), β-actin (1:2000, Santa Cruz Biotechnology, sc-47778), and then with secondary HRP-conjugated anti-mouse (1:5000, Millipore, Burlington, MA, USA, AP124P), or anti-rabbit antibodies (1:5000, Millipore, AP132P) for 1 h at room temperature. The labeled proteins were detected using Chemiluminescent Reagent (Pierce Biotechnology Inc., Rockford, IL, USA) and an LAS-4000 imaging system (Fujifilm Inc., Stanford, CT, USA). The relative intensities of protein bands, compared with that of the respective β-Actin signals, were determined using Multi Gauge software, version 3.0 (Fujifilm Inc.). Whole western blot figures were provided in the File S1.

### 2.6. Total RNA Isolation, RT-PCR, and Quantitative Real-Time PCR

Total RNA was isolated using Trizol reagent (Sigma-Aldrich, #T9424) according to the manufacturer’s protocol. RT-CPR was conducted with 3 μg of total RNA using reverse transcriptase (Thermo, Waltham, MA, USA, EP0442) by a VeritiPro™ Thermal Cycler (Applied Biosystems, Waltham, MA, USA, #A48141). Quantitative real-time PCR (qRT PCR) was performed using an SYBR Green Master Mix (Bio-Rad, Hercules, CA, USA, #1708882) with a CFX Connect Real-Time PCR Detection System (Bio-rad, #1855201). All data were obtained from three independent experiments, with each performed in triplicate. ACTB was used as an internal control for the normalization of target gene mRNA levels. Sequence information of the primers used is given in Supplementary Table S2.

### 2.7. NF-κB Luciferase Reporter Assay

NF-κB luciferase reporter assay has been described in detail in the previous publications from our group [37]. Briefly, cells were co-transfected with luciferase reporter (1 µg) containing NF-κB response element and Renilla plasmid (0.25 μg). After 24 h, cells were treated with DMSO or gefitinib for an additional 24 h. Luciferase activities were measured using a Dual-Luciferase Reporter Assay System (Promega, Madison, WI, USA) and Luminoskan Ascent luminometer (Thermo Scientific, Waltham, MA, USA). Data were obtained from three independent experiments, with each performed in triplicate and normalized with respect to Renilla luciferase activity.

### 2.8. PKC Kinase Activity Assay

Cellular PKC activities were measured using a PKC Kinase Activity Assay kit (Abcam, Cambridge, MA, UK). Cells treated with gefitinib or DMSO for 72 h were lysed in ice-cold RIPA buffer, sonicated, and centrifuged at 10,000 rpm at 4 °C for 15 min. The supernatant was assayed for PKC activity. Briefly, proteins were added to an active PKC-capturing antibody-coated 96-well plate and incubated at 30 °C for 90 min. The captured PKC proteins were treated with phospho-specific substrate antibody, washed, and then incubated with diluted anti-rabbit IgG-HRP-conjugated antibody at 25 °C for 30 min. The reacted wells were washed and incubated with TMB substrate at 25 °C for 30 min and then treated with stop solution. Absorbance was measured immediately at 450 nm using a Luminoskan microplate reader (Thermo Scientific). Data were normalized with respect to protein concentrations.

### 2.9. Measurement of PA and DAG

Cells (~1 × 10^7^) were centrifuged at 1500× *g* for 10 min, washed twice with ice-cold PBS, and resuspended in 1 mL of cold PBS. The suspended cells were homogenized in 1.5 mL methanol and mixed with 2.25 mL 1 M NaCl and 2.5 mL chloroform. The mixture was then centrifuged, and the organic phase was dried using SpeedVac. PA and DAG were detected using assay kits for DAG and PA according to the manufacturer’s instructions (Cell Biolabs, San Diego, CA, USA). Data were normalized with respect to cell numbers.

### 2.10. Apoptosis Assay Using Flow-Cytometry

Cells (5 × 10^4^) seeded in 6-well plates were treated with the indicated concentrations of gefitinib for 72 h, or etoposide (100 μM) for 2 h. The cells were trypsinized, collected by centrifugation at 900× *g* at 4 °C for 2 min, and washed twice in PBS containing 2% FBS. Apoptosis assay was performed using Annexin-V, Alexa Fluor^®^ 647 conjugate (Thermo Scientific, Carlsbad, CA, USA) according to the manufacturer’s protocol. In brief, collected cells were resuspended in 500 µL of binding buffer (Thermo Scientific, Carlsbad, CA, USA) containing 5 µL Annexin-V and propidium iodide (PI) (Sigma-Aldrich, Saint Louis, MO, USA). Fluorescence intensities of each population of 10,000 cells were measured using a BD FACSVerse system (BD Biosciences, San Jose, CA, USA).

### 2.11. Nile Red Staining

Cells seeded on Ibidi μ-slide 8 well (Ibidi, Martinsried, Germany) were washed with PBS, fixed in 4% paraformaldehyde for 20 min at room temperature, washed twice with PBS. Each well was incubated with 5 μg/mL Nile red (Sigma-Aldrich, St. Louis, MO, USA) in PBS for 10 min and washed twice with PBS. Cells were visualized under a fluorescence microscope (Nikon, Tokyo, Japan).

### 2.12. In Vivo Evaluation of Anticancer Activity in an H1650 Xenograft Model

H1650/shLPIN1 and H1650/pLKO cells (5 × 10^6^) resuspended in 100 μL of PBS were mixed with 100 μL of matrigel (Corning Inc., New York City, NY, USA, #354248) and subcutaneously injected into the right rear flank of 5-week-old female BALB/c nude mice (SLC Inc., Hamamatsu, Shizuoka, Japan). The H1650/shLPIN1 or H1650/pLKO group was randomly stratified into two or four subgroups, respectively, and treatments (gefitinib and/or LPIN inhibitor) were initiated when all mice had a mean tumor size of approximately 200 mm^3^. Each subgroup was administered 200 μL DMSO (10%, 20 μL DMSO + 180 μL PBS containing 5% tween 80) or gefitinib (30 mg/kg) and/or propranolol (10 mg/kg) intraperitoneally every 3 days for 6 weeks (*n* = 5/group). The body weight and tumor size of each mouse were measured in two dimensions using a caliper twice a week. Tumor size was calculated using the equation (w^2^ *l)/2, where l and w represent the largest and smallest dimensions in each measurement. On day 3 after the last inhibitor treatment, tumor tissues were excised and snap-frozen in liquid nitrogen for protein analysis.

### 2.13. Statistical Analysis

All the statistical data are represented as the mean ± standard deviation (SD) or standard error of the mean (SEM). The *p*-values for determining statistical significance were calculated using an unpaired two-tailed Student’s *t*-test or two-way ANOVA. Symbols used were: *, *p* < 0.05; **, *p* < 0.01; ***, *p* < 0.001; ****, *p* < 0.0001; and NS, not significant.

## 3. Results

### 3.1. LPIN1 Depletion Increases Gefitinib Sensitivity by Enhancing Apoptosis in TKI-Resistant NSCLC Cells

**Figure 1:**
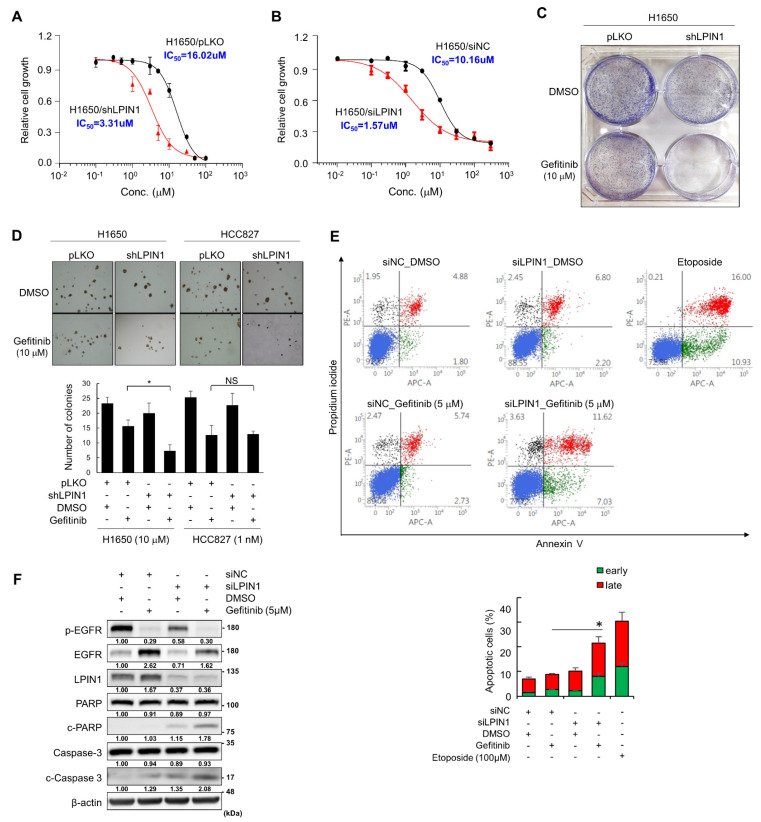
LPIN1 depletion increases gefitinib sensitivity by enhancing apoptosis in TKI-resistant NSCLC cells. (**A**) H1650 cells stably transduced with a lentiviral vector carrying shLPIN1 (H1650/shLPIN1) or shControl (H1650/pLKO) were treated with gefitinib or DMSO for 120 h. The gefitinib sensitivity of each cell line was determined using varying gefitinib concentrations. (**B**) H1650 cells were transfected with siRNAs for LPIN1 and then treated with gefitinib or DMSO for 120 h. The gefitinib sensitivity of each cell line was determined using varying gefitinib concentrations. (**C**) H1650 cells were infected with shLPIN1- or pLKO-harboring lentivirus and treated with gefitinib (10 μM) or DMSO for 10 days. Cell growth was measured by a colony formation assay after 0.5% crystal violet staining. (**D**) H1650 and HCC827 cells were infected with shLPIN1- or pLKO-harboring lentivirus and treated with gefitinib (10 μM in H1650 or 1 nM in HCC827 cells) or DMSO for 14 days. Cell growth was measured by an anchorage-independent growth assay in soft agar. (**E**) H1650 cells were transfected with siRNAs for LPIN1; treated with gefitinib (5 μM) or DMSO for 72 h; harvested; stained with Annexin-V, Alexa Fluor^®^ 647 conjugate, and PI; and analyzed via flow cytometry. Etoposide (100 μM) was used as the positive control for inducing apoptosis. (**F**) Levels of proteins including cleaved PARP and caspase-3 were analyzed via western blotting. β-Actin was used as a loading control. Values are the means ± SD of three independent experiments. Statistical significance was determined by Student’s *t*-test (*, *p* < 0.05 and NS, not significant).

From the previous genome-wide RNAi screening of TKI-resistant NSCLC H1650 cells [37], we selected LPIN1 as a factor closely related to gefitinib resistance. To study the effect of LPIN1 on gefitinib resistance in EGFR-activating mutant NSCLC cells, we determined the IC_50_ values of gefitinib in TKI-resistant H1650 cells stably transduced with LPIN1-specific or control shRNA and found that LPIN1-depleted H1650 cells exhibited an IC_50_ value approximately 5-fold lower than that of the control cells (Figure 1A). A similar effect was also observed in LPIN1-specific siRNA-treated H1650 cells, showing approximately 7.5-fold lower IC_50_ value than that of the control siRNA-transfected H1650 cells (Figure 1B). In contrast, LPIN1-specific siRNA-treated TKI-sensitive HCC827 and PC-9 cells did not alter, or very slightly altered, the IC_50_ value (Supplementary Figure S1). In addition, colony formation (Figure 1C) and anchorage-independent growth (Figure 1D) were strikingly reduced in LPIN1-depleted H1650 cells by treatment of gefitinib, contrary to no further reduction in anchorage-independent growth of LPIN1-depleted TKI-sensitive HCC827 cells by treatment of gefitinib (Figure 1D). These results demonstrate that LPIN1 depletion increases sensitivity to gefitinib in H1650 cells.

To determine whether these results were due to cell death, gefitinib-resistant H1650 cells transiently transfected with siLPIN1 or control siRNA (siNC) were double-stained with Alexa Fluor^®^ 647-conjugated Annexin-V and PI and subjected to flow cytometry analysis. LPIN1 knockdown increased apoptosis (early and late apoptosis) in gefitinib-treated H1650 cells (Figure 1E). In addition, we identified a further increase in the levels of cleaved-PARP and -caspase 3 in LPIN1-depleted cells upon gefitinib treatment (Figure 1F). These results demonstrate that LPIN1 induces gefitinib resistance by blocking drug-induced apoptosis in H1650 cells.

### 3.2. LPIN1 Promotes PKCδ Activation by Increasing DAG Production upon Gefitinib Treatment in TKI-Resistant Cell

**Figure 2:**
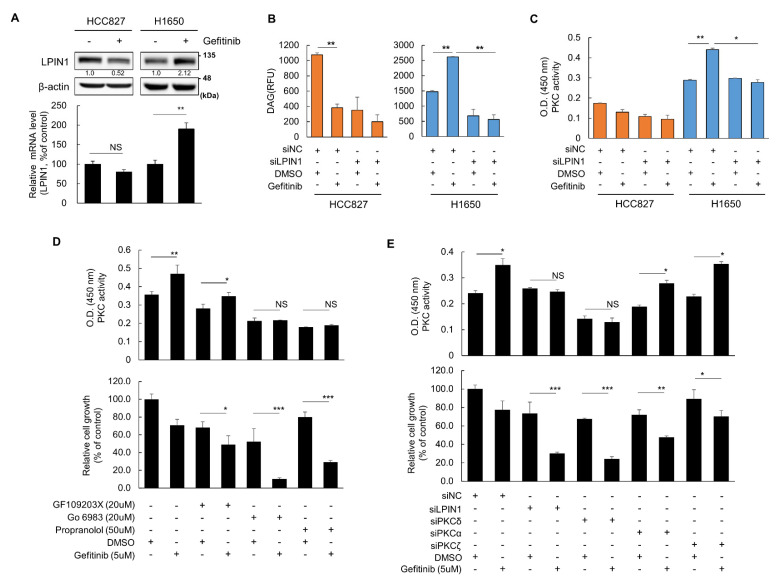
LPIN1 promotes PKCδ activation by increasing DAG production upon gefitinib treatment in TKI-resistant cells. (**A**) The effect of gefitinib treatment on LPIN1 expression was measured. The mRNA and protein levels in the HCC827 and H1650 cells treated or untreated with gefitinib were determined via qRT-PCR or western blotting, respectively. (**B**,**C**) HCC827 and H1650 cells were transfected with siLPIN1 or siControl (siNC). (**B**) DAG levels or (**C**) total PKC activity was determined after gefitinib (5 μM) or DMSO treatment for 24 h. (**D**) H1650 cells were pretreated with PKCα, PCKβ, PKCδ, or PKCγ inhibitors (GF109203X (20 μM S7208) or Go 6983 (20 μM S2911)) or LPIN inhibitor (50 μM propranolol) for 2 h, treated with 5 μM gefitinib, and subjected to PKC activity and cell counting assays. (**E**) H1650 cells were transfected with siRNAs for LPIN1, PKCα, PKCδ and PKCζ individually or as a mixture, treated with gefitinib (5 μM) or DMSO for 72 h, and subjected to PKC activity and cell counting assays. Values are the mean ± SD of three independent experiments. Statistical significance was determined by (**A**,**D**,**E**) Student’s *t*-test or (**B**,**C**) two-way ANOVA (*, *p* < 0.05; **, *p* < 0.01; ***, *p* < 0.001; and NS, not significant).

LPIN1 can potentially affect the cellular levels of PA and DAG, the substrate and product of LPIN1, respectively. We determined the cellular levels of PA and DAG in gefitinib-sensitive HCC827 and gefitinib-resistant H1650 cells. Interestingly, unlike gefitinib-sensitive HCC827 cells, cellular DAG levels did not decrease in gefitinib-resistant H1650 cells, and some even increased following gefitinib treatment (Supplementary Figure S2). Cellular PA levels were not significantly changed in both EGFR-mutant NSCLC cells (Supplementary Figure S3). Consistent with this, the expression of LPIN1 mRNA and protein changed accordingly with the changes in DAG levels; both mRNA and protein expression levels were reduced in HCC827 cells but induced in H1650 cells following gefitinib treatment (Figure 2A). The gefitinib-mediated LPIN1 reduction was validated in the other TKI-sensitive PC-9 cells (Supplementary Figure S4). This suggests that LPIN1 mainly regulates differential changes in cellular DAG levels following gefitinib treatment in both cell lines. Additionally, we found that LPIN1 knockdown clearly decreased both the basal and gefitinib treatment-induced DAG contents and increased PA contents in gefitinib-resistant H1650 NSCLC cells (Figure 2B and Figure S5). Therefore, the gefitinib-induced LPIN1 triggers the accumulation of intracellular DAG in gefitinib-resistant H1650 cells.

Knowing that DAG induces PKC activation, we compared the relationship between LPIN1 expression and PKC activation in gefitinib-sensitive and gefitinib-resistant lung cancer cells. Consistent with the result showing a higher content of DAG in H1650 than in HCC827 cells (Figure 2B), the basal level of PKC activity was higher in the gefitinib-resistant H1650 cells. Upon gefitinib treatment, PKC activity increased only in H1650 cells, similar to the increase in DAG levels, and LPIN1 depletion abrogated the induction of PKC activation (Figure 2C). This implies that LPIN1 is a key factor responsible for altered PKC activation in gefitinib-resistant cancer cells.

To assess the potential association between altered PKC activation and gefitinib resistance, we examined the relationship between PKC activity and cell viability using pharmacological and RNAi approaches. Although treatment with PKC inhibitors suppressed basal PKC activity, only the PKC inhibitor Go 6983 completely aborted the induction of PKC activation following gefitinib treatment (Figure 2D, upper panel). At the same time, the sensitivity of H1650 cells to gefitinib remarkably increased when gefitinib was co-treated with Go 6983 (Figure 2D, lower panel), suggesting that PKCδ is responsible for gefitinib-dependent PKC activation and drug resistance in H1650 cells. The LPIN inhibitor propranolol treatment also aborted the induction of PKC activation and significantly increased gefitinib sensitivity in H1650 cells following gefitinib treatment (Figure 2D). These results demonstrate that LPIN1 is responsible for drug resistance in H1650 cells by modulating PKC activation, particularly PKCδ. To further determine the involvement of the PKCδ subtype, we performed RNAi experiments using subtype-specific siRNAs against PKC (Supplementary Figure S6) and found that only siRNA of PKCδ significantly blocked gefitinib-mediated PKC activation and inhibited cell growth, similar to the effects exerted by siLPIN1 (Figure 2E). Therefore, LPIN1 promotes DAG production and induces PKCδ activation, which is essential for gefitinib resistance in NSCLC cells.

### 3.3. Activated NF-κB Signaling and LD Formation through LPIN1-Mediated Activation of PKCδ Are Responsible for Gefitinib Resistance in H1650 Cells

**Figure 3:**
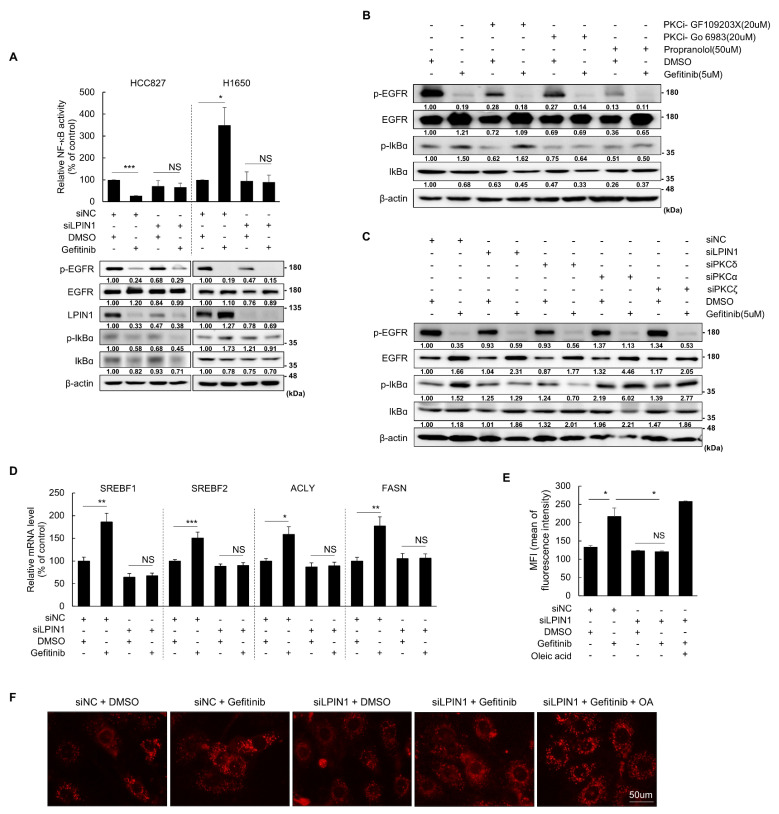
NF-κB signaling and LD formation are activated in an LPIN1-dependent manner in gefitinib-resistant H1650 cells. (**A**) H1650/siLPIN1, H1650/siNC, HCC827/siLPIN1, or HCC827/siNC cells transfected with NF-κB luciferase reporter plasmid were treated with gefitinib (5 μM) or DMSO for 24 h and then harvested to determine NF-κB activity (upper panel) and p-IκBα expression levels (lower panel). (**B**) H1650 cells were pretreated with PKCα, β, or γ inhibitors (GF109203X (20 μM, S7208) or Go 6983 (20 μM, S2911)) or LPIN inhibitor (50 μM propranolol) for 2 h, treated with 5 μM gefitinib, and subjected to western blot analysis to determine p-IκBα expression levels. (**C**) H1650 cells were transfected with siRNAs for LPIN1, PKCα, PKCδ and PKCζ individually, treated with gefitinib (5 μM) or DMSO for 24 h, and subjected to western blot analysis to determine p-IκBα expression levels. (**D**) H1650 cells were transfected with siRNAs for LPIN1 and then treated with gefitinib (5 μM) or DMSO for 24 h. qRT-PCR was performed to determine the mRNA expression of the indicated genes. (**E**,**F**) H1650 cells were transfected with siRNAs for LPIN1 and treated with gefitinib (5 μM) or DMSO for 24 h. The cells were subjected to € flow cytometry to determine lipid droplet production or (**F**) Nile red staining to visualize lipid droplets under a fluorescence microscope. Oleic acid (10 μM) was used as the positive control of lipid droplet production. Values are the mean ± SD of three independent experiments. Statistical significance was determined by (**A**,**D**) Student’s *t*-test € (**E**) two-way ANOVA (*, *p* < 0.05; **, *p* < 0.01; ***, *p* < 0.001; and NS, not significant).

To validate the PKCδ-mediated function of LPIN1 in gefitinib resistance, we investigated two downstream effects of PKC function, NF-κB signaling and lipogenesis [22,23,29], which play important roles in tumorigenesis and drug resistance by providing pro-survival signals to cancer cells [26,27,28,29]. First, we examined whether NF-κB signaling is associated with LPIN1-dependent gefitinib resistance in H1650 cells. Gefitinib-treated TKI-sensitive HCC827 cells showed decreased LPIN1 expression and NF-κB activity (Figure 3A). However, gefitinib treatment increased LPIN1 expression and NF-κB activity, and LPIN1 depletion reduced gefitinib-induced NF-κB activity and p-IκBα levels in TKI-resistant H1650 cells (Figure 3A). To further validate the association between NF-κB activation and LPIN1-induced PKC activity, we examined p-IκBα levels using pharmacological and RNAi approaches. Although treatment with PKC inhibitors suppressed basal p-IκBα levels, the PKC inhibitor Go 6983, but not the PKC inhibitor GF109203X, prevented p-IκBα level induction following gefitinib treatment (Figure 3B), suggesting that PKCδ is responsible for gefitinib-dependent NF-κB activation in H1650 cells. Propranolol treatment also aborted the induction of p-IκBα levels in H1650 cells following gefitinib treatment (Figure 3B). In addition, we performed RNAi experiments using subtype-specific siRNAs against PKC and found that only siRNA of PKCδ significantly blocked gefitinib-mediated induction of p-IκBα levels, similar to the effects exerted by siLPIN1 (Figure 3C). This result demonstrates that LPIN1 regulates the activation of NF-κB signaling through activation of PKCδ in gefitinib-treated H1650 cells.

Next, we investigated the expression of genes involved in lipid synthesis as targets of the PKC signaling pathway. Consistent with PKC activation, expression of lipogenic genes, including SREBFs, ATP citrate lyase (ACLY), and fatty acid synthase (FASN), was significantly increased by gefitinib treatment in gefitinib-resistant H1650 cells, and the up-regulated expression was remarkably inhibited by LPIN1 depletion (Figure 3D). LD formation was significantly increased following gefitinib treatment in gefitinib-resistant H1650 cells in a LPIN1 expression-dependent manner (Figure 3E,F). These results demonstrate that LPIN1 is a major regulator of gefitinib-induced survival signaling that involves PKC activation-mediated NF-κB activation and LD formation in TKI-resistant H1650 cells.

### 3.4. Gefitinib Resistance Is Highly Dependent on DAG Content in H1650 Cells

**Figure 4:**
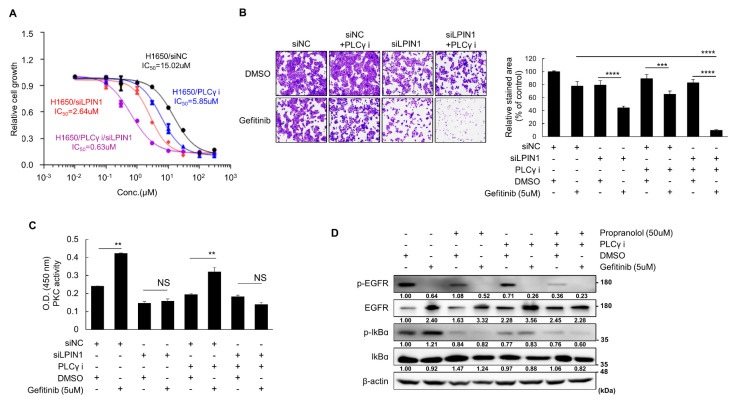
Gefitinib resistance is highly dependent on the total amount of DAG in H1650 cells. (**A**) H1650 cells were transfected with siRNAs for LPIN1 and treated with a PLCγ inhibitor (5 μM) or a vehicle in combination with varying gefitinib concentrations. The gefitinib sensitivity of each cell line was determined using varying gefitinib concentrations. (**B**) H1650 cells were transfected with siRNAs for LPIN1 and treated with the PLCγ inhibitor (5 μM) or vehicle in combination with gefitinib (5 μM) or DMSO for 96 h; cell growth was measured using 0.5% crystal violet staining. (**C**) H1650 cells were transfected with siRNAs for LPIN1, treated with PLCγ inhibitor (5 μM) or vehicle in combination with gefitinib (5 μM) or DMSO for 72 h, and harvested to detect their PKC activity. (**D**) H1650 cells were treated with propranolol (50 μM), PLCγ inhibitor (5 μM) or vehicle in combination with gefitinib (5 μM) or DMSO for 24 h and harvested to determine p-IκBα expression levels. Values are the mean ± SD of three independent experiments. Statistical significance was determined by (**B**) two-way ANOVA or (**C**) Student’s *t*-test (**, *p* < 0.01; ***, *p* < 0.001; ****, *p* < 0.0001; and NS, not significant).

Cellular DAG is produced by LPIN1, which dephosphorylates PA in de novo synthesis of lipids, and by PLC, which cleaves phosphatidylinositol 4,5-bisphosphate (PIP_2_) to generate DAG and inositol 1,4,5-triphosphate (IP3) [38]. DAG, which is generated by PLCγ docked in EGFR, induces survival signaling in TKI-resistant cancer cells by activating PKCδ [29]. Therefore, to compare the effects of LPIN1 and PLCγ on gefitinib sensitivity, we treated H1650 cells with PLCγ inhibitor U73122 or LPIN1-specific siRNA. Gefitinib sensitivity of H1650 cells increased more with LPIN1 knockdown (approximately 5-fold) than PLCγ inhibition (approximately 2.5-fold), and their co-treatment synergistically increased the sensitivity by more than 20-fold (Figure 4A). We validated these results through a clonogenic assay that showed a slightly stronger effect of LPIN1 knockdown than PLCγ inhibition and a synergetic effect of their co-treatment (Figure 4B). In addition, we found that LPIN1 knockdown clearly inhibited both the basal and gefitinib treatment-induced activities of PKC, while the PLCγ inhibitor U73122 only marginally inhibited the PKC activities, in H1650 cells (Figure 4C). Moreover, LPIN1 inhibition more clearly reduced gefitinib-induced p-IκBα expression than PLCγ inhibition did (Figure 4D). Therefore, LPIN1-mediated DAG production is important to acquire gefitinib resistance in H1650 cells, and blocking LPIN1 and PLC simultaneously may be effective in treating drug-resistant cancers.

### 3.5. Patient-Derived EGFR-Mutant NSCLC Cells Show LPIN1-Dependent Gefitinib Resistance through Induction of PKCδ Signaling

**Figure 5:**
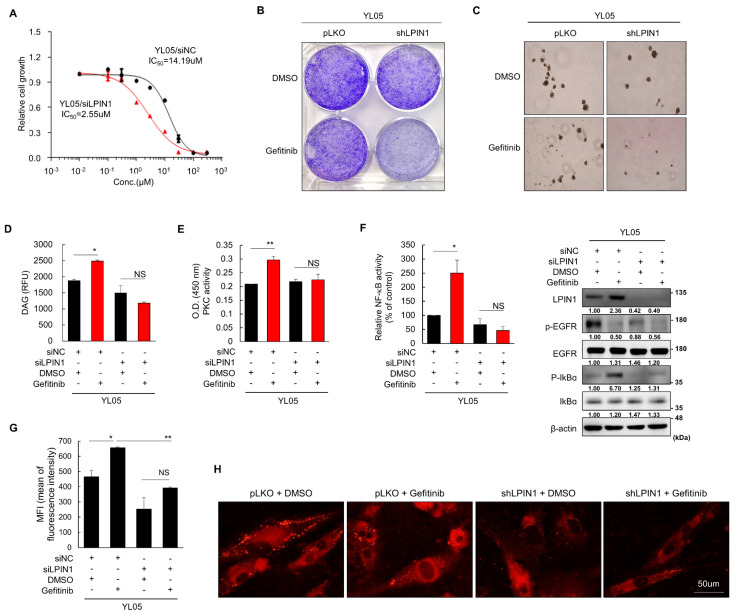
Patient-derived EGFR mutant NSCLC cells show LPIN1-dependent gefitinib resistance via PKCδ signaling induction. (**A**) Gefitinib-resistant lung cancer PDCs (YL05) were transfected with LPIN1- or NC-siRNA and treated with gefitinib (5 μM) or DMSO for (**A**) 120 h. Cell growth was measured by an MTT assay. (**B**,**C**) Gefitinib-resistant lung cancer PDCs were infected with shLPIN1- or pLKO-harboring lentivirus and treated with gefitinib (5 μM) or DMSO for (**B**) 10 days, and (**C**) 14 days. Cell growth was measured by (**B**) a colony formation assay after 0.5% crystal violet staining, or (**C**) an anchorage-independent growth assay in soft agar. (**D**,**E**) YL05 cells were transfected with siLPIN1 or siNC and treated with gefitinib (5 μM) or DMSO (**D**) for 24 h and harvested to determine the amount of diacylglycerol, (**E**) for 72 h and harvested to detect PKC activity. (**F**) YL05/siLPIN1 or YL05/siNC cells transfected with an NF-κB luciferase reporter plasmid were treated with gefitinib (5 μM) or DMSO for 24 h and harvested to determine their NF-κB activity and p-IκBα expression level. (**G**,**H**) LPIN1-depleted YL05 cells by (**G**) siRNA or (**H**) shRNA were treated with gefitinib (5 μM) or DMSO for 24 h. The cells were subjected to (**G**) flow cytometry to determine lipid droplet production or (**H**) Nile red staining to visualize lipid droplets with a fluorescence microscope. Values are the mean ± SD of three independent experiments. Statistical significance was determined by (**D**–**F**) Student’s *t*-test or (**G**) two-way ANOVA (*, *p* < 0.05; **, *p* < 0.01; and NS, not significant).

The involvement of LPIN1 in gefitinib resistance was further validated using lung cancer patient-derived cells (PDCs) YL05 [37]. Consistent with the results of H1650 cells, LPIN1 depletion significantly sensitized YL05 cells to gefitinib treatment (Figure 5A). Both anchorage-dependent (clonogenic assay) and anchorage-independent (soft-agar assay) growth of PDCs treated with gefitinib were remarkably decreased when LPIN1 was knocked down (Figure 5B,C). In addition, gefitinib treatment in YL05 PDCs increased DAG production, followed by subsequent activation of PKC (Figure 5D–H), NF-κB, and LD formation (Figure 5D–H). Furthermore, LPIN1 silencing aborted gefitinib-dependent induction of DAG production, PKC activation, NF-κB activation, and LD formation in YL05 PDC cells (Figure 5D–H). These results suggest the clinical relevance of the LPIN1/DAG/PKCδ axis in inducing TKI resistance in lung cancer cells.

### 3.6. Loss of LPIN1 Expression Sensitizes EGFR-Mutant NSCLC Cells to Gefitinib In Vivo

**Figure 6:**
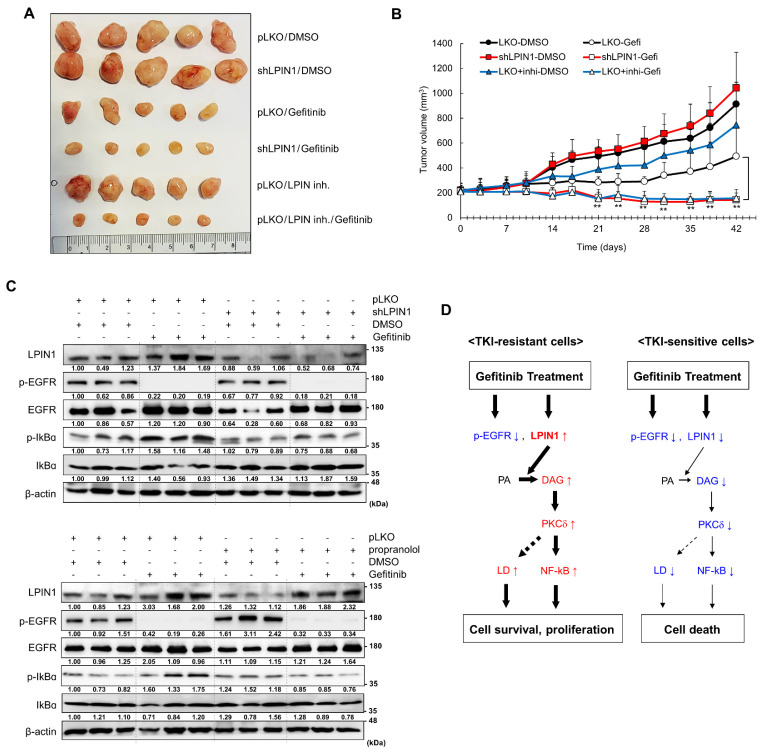
Loss of LPIN1 expression sensitizes EGFR-mutant NSCLC cells to gefitinib in vivo. (**A**,**B**) Mice bearing H1650/shLPIN1 or H1650/pLKO tumor xenografts were subcutaneously injected with 30 mg/kg gefitinib or/and 10 mg/kg LPIN inhibitor, propranolol or vehicle twice a week (*n* = 5/group), and tumor growth was monitored for 6 weeks. (**A**) Tumors resected from each group at the end of experiments and profiles of tumor growth during 6 weeks of gefitinib treatment. (**B**) For each treatment group, data are presented as the mean tumor volume (mm^3^) ± SEM. (**C**) The protein expression levels in the resected tumors were analyzed via western blotting. β-Actin was used as a loading control. (**D**) Schematic diagram of LPIN1-mediated gefitinib resistance. (red, ↑: increase of expression, amount or activity; blue, ↓: decrease of expression, amount or activity) Statistical significance was determined by Student’s *t*-test (**, *p* < 0.01).

Finally, we examined whether in vitro suppression of gefitinib resistance by LPIN1 depletion could be recapitulated in vivo using H1650 cells xenografted into nude mice. H1650-derived tumors expressing shLPIN1 or pLKO were treated twice a week with an intraperitoneal injection of gefitinib (30 mg/kg) and/or the LPIN1 inhibitor, propranolol (10 mg/kg), or vehicle. Consistent with in vitro results, LPIN1 knockdown or inhibitor treatment rarely affected tumor growth compared with the control group (Figure 6A,B). However, gefitinib treatment combined with LPIN1 depletion or inhibition, significantly inhibited tumor growth compared to treatment with gefitinib alone. Western blot analysis of resected tumors confirmed that LPIN1 expression and NF-κB activity (IκBα phosphorylation) were clearly increased by gefitinib treatment, and an increased IκBα phosphorylation was effectively suppressed by gefitinib treatment combined with LPIN1 depletion or inhibition (Figure 6C). These in vivo data further support the roles of gefitinib-induced LPIN1 expression and NF-κB activation in cell survival (Figure 6D) and the importance of LPIN1 as a potential target to prevent gefitinib resistance in EGFR-mutant NSCLC.

## 4. Discussion

In this study, we propose LPIN1 as a factor regulating gefitinib resistance in EGFR-mutant lung cancer cells, which upon depletion, results in synthetic lethality with gefitinib treatment. Altered lipid metabolism in cancer modulates drug resistance. The activation of glucosylceramide synthase, which converts ceramide to glucosylceramide, stimulates cell growth and DNA synthesis to drive cancer cell resistance to chemotherapy [39]. Upregulation of lipogenesis and associated LD accumulation elicits a cytoprotective response to oxidative stress by decreasing reactive oxygen species-mediated toxicity, thereby increasing cancer cell survival [40]. In addition, gefitinib treatment induces the production of LDs in drug-resistant cancer cells but reduces it in drug-sensitive cancer cells [19]. LPINs are lipogenic genes that produce DAG, an important precursor for neutral lipid triglycerides deposited in LDs. However, nuclear LPIN1, whose localization is changed in a phosphorylation-dependent manner [41], inhibits cellular lipogenesis by sequestering SREBPs from the nucleus [31]. mTORC1 phosphorylates and blocks the nuclear localization of LPIN1, allowing the nuclear localization of SREBPs and, consequently, inducing lipogenic genes [31]. Therefore, it is presumed that mTORC1 is activated in drug-resistant cells and phosphorylates LPIN1, resulting in cytosolic LPIN1-dependent LD formation. mTORC1 has been reported to become activated following gefitinib treatment in TKI-resistant cells and promote resistance to chemotherapy and targeted cancer drugs [42,43]. We observed that LPIN1 depletion mitigated gefitinib-induced LD production in drug-resistant cancer cells. In addition, interestingly, lipogenic genes, including SREBFs, ACLY and FASN, were reduced by LPIN1 knockdown, suggesting that LPIN1 is not a passive lipogenic gene but a driver gene that actively induces alterations in lipid metabolism in TKI-resistant cancer cells.

Unlike TKI-sensitive HCC827 cells, the intracellular level of LPIN1 was transcriptionally induced by gefitinib treatment in TKI-resistant H1650 and patient-derived TKI-resistant lung cancer cells. The expression of lipogenic genes, including LPIN1, is induced by mTORC2, probably through PPARγ [44,45]. In addition, mTORC2 is activated in drug-resistant cancer cells through an undefined mechanism [46,47]. We also found that the effect of gefitinib treatment on LPIN1 expression completely disappeared when the rapamycin-insensitive companion of the mammalian target of rapamycin (RICTOR) was knocked down by transfection with siRICTOR (Supplementary Figure S7). These results suggest a possible mechanism for LPIN1 induction in TKI-resistant lung cancer cells mediated by mTORC2. Although the mechanisms of simultaneous activation of mTORC1 and mTORC2 in TKI-resistant cells remain to be elucidated, gefitinib-induced LPIN1 expression may mediate activation of PKCs, an upstream signal of SREBPs [22], by increasing DAG production in TKI-resistant cells, and this transfers the cells into a lipogenic state, consequently inducing LD formation.

PKC is activated by DAG in two ways: acute activation by DAG produced by PLC in the cell membrane and long-term activation by DAG produced by LPIN in cytosolic vesicle membrane [48]. The importance of DAG-mediated PKC activation in gefitinib resistance of cancer cells has recently been reported [29]. According to this study, PLCγ is docked to Y1173 of EGFR, which is still phosphorylated by gefitinib treatment, and cleaves PIP_2_ to produce IP_3_ and DAG. DAG induces the activation of PKCδ and cancer cell survival. Our results also demonstrated that cancer cells acquire resistance to gefitinib by increasing DAG, which is produced by LPIN1. Furthermore, concomitant inhibition of both LPIN1 and PLCγ to further block DAG production dramatically increased the gefitinib sensitivity of the resistant cells. Therefore, the total amount of DAG is crucial for cancer progression in gefitinib-resistant cells.

Together with the observations in the mouse xenograft model, our results suggest that LPIN1 targeting is a good strategy for treating gefitinib-resistant NSCLC, which coincidently modulates survival factors, including PKC-NF-kB pathway signaling and altered lipid metabolism, such as LD formation. Multiple resistance mechanisms drive drug resistance to EGFR-TKIs, and these can frequently co-occur in patients, suggesting that it can be extremely difficult to completely overcome all resistance mechanisms in patients [4,5,6,7,8,9]. Therefore, defining novel mechanisms or targets that regulate resistance will increase the potential of cancer treatment. In this regard, the therapeutic potential of targeting LPIN1 in EGFR-mutant NSCLC requires further investigation.

## 5. Conclusions

In this study, we investigated whether LPIN1 could regulate gefitinib resistance in EGFR-mutant NSCLC cells. We defined the molecular mechanisms underlying the LPIN1-mediated activation of PKCδ pathway signaling. Our results demonstrated that gefitinib treatment induced LPIN1 expression and increased DAG concentration in TKI-resistant H1650 cells. It also activated PKCδ-NF-κB pathway signaling and LD formation in an LPIN1-dependent manner, resulting in cancer cell survival. Conversely, LPIN1 depletion and pharmaceutical inhibition clearly attenuated in vitro and in vivo resistance to gefitinib in EGFR-mutant NSCLC. These results were recapitulated in a patient-derived EGFR-mutant NSCLC cell line. Therefore, LPIN1 is a potential factor regulating gefitinib resistance in EGFR-activating mutant NSCLC cells. Moreover, co-treatment with TKIs and LPIN1 inhibitors represents a promising therapeutic approach to overcoming TKI resistance in NSCLC patients.

## Data Availability

The data presented in this study are available on request from the corresponding author.

## Supplementary Materials

The following supporting information can be downloaded at: https://www.mdpi.com/article/10.3390/cancers14092222/s1, Table S1: shRNA and siRNA sequences; Table S2: Primer sequences; Figure S1: Determination of IC50 in HCC827 and PC-9 cells; Figure S2: Determination of the amount of DAG in H1650 and HCC827 cells; Figure S3: Determination of the amount of PA in H1650 and HCC827 cells; Figure S4: Determination of the protein levels of LPIN1 in PC-9 cells; Figure S5: Determination of the amount of PA in H1650 and HCC827 cells transfected with siRNAs for LPIN1; Figure S6: Determination of the levels of LPIN1, PRKCA, PRKCD, PRKCZ mRNA in H1650 cells transfected with siRNA for themselves; Figure S7: Determination of the levels of LPIN1and RICTOR mRNA in H1650 cells transfected with siRNAs for RICTOR; File S1: Whole western blots.

## References

[1] Vescio R.A., Connors K.M., Bordin G.M., Robb J.A., Youngkin T., Umbreit J.N., Hoffman R.M. (1990). The distinction of small cell and non-small cell lung cancer by growth in native-state histoculture. Cancer Res..

[2] Torre L.A., Bray F., Siegel R.L., Ferlay J., Lortet-Tieulent J., Jemal A. (2015). Global cancer statistics, 2012. CA Cancer J. Clin..

[3] Bhuiyan S., Siddiqui R.S., Zirkiyeva M., Agladze M., Bashir T. (2021). A Rare Case of Small Cell Lung Cancer with an Epidermal Growth Factor Receptor Mutation and Its Response to Osimertinib. Cureus.

[4] Stewart E.L., Tan S.Z., Liu G., Tsao M.S. (2015). Known and putative mechanisms of resistance to EGFR targeted therapies in NSCLC patients with EGFR mutations-a review. Transl. Lung Cancer Res..

[5] Phuchareon J., McCormick F., Eisele D.W., Tetsu O. (2015). EGFR inhibition evokes innate drug resistance in lung cancer cells by preventing Akt activity and thus inactivating Ets-1 function. Proc. Natl. Acad. Sci. USA.

[6] Leonetti A., Sharma S., Minari R., Perego P., Giovannetti E., Tiseo M. (2019). Resistance mechanisms to osimertinib in EGFR-mutated non-small cell lung cancer. Br. J. Cancer.

[7] Pao W., Miller V.A., Politi K.A., Riely G.J., Somwar R., Zakowski M.F., Kris M.G., Varmus H. (2005). Acquired resistance of lung adenocarcinomas to gefitinib or erlotinib is associated with a second mutation in the EGFR kinase domain. PLoS Med..

[8] Bean J., Brennan C., Shih J.Y., Riely G., Viale A., Wang L., Chitale D., Motoi N., Szoke J., Broderick S. (2007). MET amplification occurs with or without T790M mutations in EGFR mutant lung tumors with acquired resistance to gefitinib or erlotinib. Proc. Natl. Acad. Sci. USA.

[9] Mukohara T. (2011). Mechanisms of resistance to anti-human epidermal growth factor receptor 2 agents in breast cancer. Cancer Sci..

[10] Kroemer G., Pouyssegur J. (2008). Tumor cell metabolism: Cancer’s Achilles’ heel. Cancer Cell.

[11] Martinez-Reyes I., Chandel N.S. (2021). Cancer metabolism: Looking forward. Nat. Rev. Cancer.

[12] Zhu J., Thompson C.B. (2019). Metabolic regulation of cell growth and proliferation. Nat. Rev. Mol. Cell Biol..

[13] Munir R., Lisec J., Swinnen J.V., Zaidi N. (2019). Lipid metabolism in cancer cells under metabolic stress. Br. J. Cancer.

[14] Koundouros N., Poulogiannis G. (2020). Reprogramming of fatty acid metabolism in cancer. Br. J. Cancer.

[15] Bian X., Liu R., Meng Y., Xing D., Xu D., Lu Z. (2021). Lipid metabolism and cancer. J. Exp. Med..

[16] Feng W.W., Kurokawa M. (2020). Lipid metabolic reprogramming as an emerging mechanism of resistance to kinase inhibitors in breast cancer. Cancer Drug Resist..

[17] Li Z., Liu H., Luo X. (2020). Lipid droplet and its implication in cancer progression. Am. J. Cancer Res..

[18] Jin C., Yuan P. (2020). Implications of lipid droplets in lung cancer: Associations with drug resistance. Oncol. Lett..

[19] Huang Q., Wang Q., Li D., Wei X., Jia Y., Zhang Z., Ai B., Cao X., Guo T., Liao Y. (2019). Co-administration of 20(S)-protopanaxatriol (g-PPT) and EGFR-TKI overcomes EGFR-TKI resistance by decreasing SCD1 induced lipid accumulation in non-small cell lung cancer. J. Exp. Clin. Cancer Res..

[20] Lettiero B., Inasu M., Kimbung S., Borgquist S. (2018). Insensitivity to atorvastatin is associated with increased accumulation of intracellular lipid droplets and fatty acid metabolism in breast cancer cells. Sci. Rep..

[21] Han J., Kaufman R.J. (2016). The role of ER stress in lipid metabolism and lipotoxicity. J. Lipid Res..

[22] Schmitz-Peiffer C. (2013). The tail wagging the dog--regulation of lipid metabolism by protein kinase C. FEBS J..

[23] Frangioudakis G., Burchfield J.G., Narasimhan S., Cooney G.J., Leitges M., Biden T.J., Schmitz-Peiffer C. (2009). Diverse roles for protein kinase C delta and protein kinase C epsilon in the generation of high-fat-diet-induced glucose intolerance in mice: Regulation of lipogenesis by protein kinase C delta. Diabetologia.

[24] Ohno S., Akita Y., Hata A., Osada S., Kubo K., Konno Y., Akimoto K., Mizuno K., Saido T., Kuroki T. (1991). Structural and functional diversities of a family of signal transducing protein kinases, protein kinase C family; two distinct classes of PKC, conventional cPKC and novel nPKC. Adv. Enzym. Regul..

[25] Kolczynska K., Loza-Valdes A., Hawro I., Sumara G. (2020). Diacylglycerol-evoked activation of PKC and PKD isoforms in regulation of glucose and lipid metabolism: A review. Lipids Health Dis..

[26] Chen Z., Forman L.W., Williams R.M., Faller D.V. (2014). Protein kinase C-delta inactivation inhibits the proliferation and survival of cancer stem cells in culture and in vivo. BMC Cancer.

[27] Sadeghi M.M., Salama M.F., Hannun Y.A. (2021). Protein Kinase C as a Therapeutic Target in Non-Small Cell Lung Cancer. Int. J. Mol. Sci..

[28] He S., Li Q., Huang Q., Cheng J. (2022). Targeting Protein Kinase C for Cancer Therapy. Cancers.

[29] Lee P.C., Fang Y.F., Yamaguchi H., Wang W.J., Chen T.C., Hong X., Ke B., Xia W., Wei Y., Zha Z. (2018). Targeting PKCdelta as a Therapeutic Strategy against Heterogeneous Mechanisms of EGFR Inhibitor Resistance in EGFR-Mutant Lung Cancer. Cancer Cell.

[30] Reue K., Wang H. (2019). Mammalian lipin phosphatidic acid phosphatases in lipid synthesis and beyond: Metabolic and inflammatory disorders. J. Lipid Res..

[31] Peterson T.R., Sengupta S.S., Harris T.E., Carmack A.E., Kang S.A., Balderas E., Guertin D.A., Madden K.L., Carpenter A.E., Finck B.N. (2011). mTOR complex 1 regulates lipin 1 localization to control the SREBP pathway. Cell.

[32] Finck B.N., Gropler M.C., Chen Z., Leone T.C., Croce M.A., Harris T.E., Lawrence J.C., Kelly D.P. (2006). Lipin 1 is an inducible amplifier of the hepatic PGC-1alpha/PPARalpha regulatory pathway. Cell Metab..

[33] Zhang P., Takeuchi K., Csaki L.S., Reue K. (2012). Lipin-1 phosphatidic phosphatase activity modulates phosphatidate levels to promote peroxisome proliferator-activated receptor gamma (PPARgamma) gene expression during adipogenesis. J. Biol. Chem..

[34] Kim J.Y., Kim G., Lim S.C., Choi H.S. (2016). LPIN1 promotes epithelial cell transformation and mammary tumourigenesis via enhancing insulin receptor substrate 1 stability. Carcinogenesis.

[35] Brohee L., Demine S., Willems J., Arnould T., Colige A.C., Deroanne C.F. (2015). Lipin-1 regulates cancer cell phenotype and is a potential target to potentiate rapamycin treatment. Oncotarget.

[36] Fan X., Weng Y., Bai Y., Wang Z., Wang S., Zhu J., Zhang F. (2018). Lipin-1 determines lung cancer cell survival and chemotherapy sensitivity by regulation of endoplasmic reticulum homeostasis and autophagy. Cancer Med..

[37] Cho J.H., You Y.M., Yeom Y.I., Lee D.C., Kim B.K., Won M., Cho B.C., Kang M., Park S., Yang S.J. (2018). RNF25 promotes gefitinib resistance in EGFR-mutant NSCLC cells by inducing NF-kappaB-mediated ERK reactivation. Cell Death Dis..

[38] Kadamur G., Ross E.M. (2013). Mammalian phospholipase C. Annu. Rev. Physiol..

[39] Liu Y.Y., Hill R.A., Li Y.T. (2013). Ceramide glycosylation catalyzed by glucosylceramide synthase and cancer drug resistance. Adv. Cancer Res..

[40] Rysman E., Brusselmans K., Scheys K., Timmermans L., Derua R., Munck S., Van Veldhoven P.P., Waltregny D., Daniels V.W., Machiels J. (2010). De novo lipogenesis protects cancer cells from free radicals and chemotherapeutics by promoting membrane lipid saturation. Cancer Res..

[41] Harris T.E., Huffman T.A., Chi A., Shabanowitz J., Hunt D.F., Kumar A., Lawrence J.C. (2007). Insulin controls subcellular localization and multisite phosphorylation of the phosphatidic acid phosphatase, lipin 1. J. Biol. Chem..

[42] Ilagan E., Manning B.D. (2016). Emerging role of mTOR in the response to cancer therapeutics. Trends Cancer.

[43] Mossmann D., Park S., Hall M.N. (2018). mTOR signalling and cellular metabolism are mutual determinants in cancer. Nat. Rev. Cancer.

[44] Guo Z., Zhao K., Feng X., Yan D., Yao R., Chen Y., Bao L., Wang Z. (2019). mTORC2 Regulates Lipogenic Gene Expression through PPARgamma to Control Lipid Synthesis in Bovine Mammary Epithelial Cells. Biomed. Res. Int..

[45] Schadinger S.E., Bucher N.L., Schreiber B.M., Farmer S.R. (2005). PPARgamma2 regulates lipogenesis and lipid accumulation in steatotic hepatocytes. Am. J. Physiol. Endocrinol. Metab..

[46] Fei S.J., Zhang X.C., Dong S., Cheng H., Zhang Y.F., Huang L., Zhou H.Y., Xie Z., Chen Z.H., Wu Y.L. (2013). Targeting mTOR to overcome epidermal growth factor receptor tyrosine kinase inhibitor resistance in non-small cell lung cancer cells. PLoS ONE.

[47] Chiang C.T., Demetriou A.N., Ung N., Choudhury N., Ghaffarian K., Ruderman D.L., Mumenthaler S.M. (2018). mTORC2 contributes to the metabolic reprogramming in EGFR tyrosine-kinase inhibitor resistant cells in non-small cell lung cancer. Cancer Lett..

[48] Gallegos L.L., Newton A.C. (2008). Spatiotemporal dynamics of lipid signaling: Protein kinase C as a paradigm. IUBMB Life.

