# Supplementary material for: LPIN1 Induces Gefitinib Resistance in EGFR Inhibitor-Resistant Non-Small Cell Lung Cancer Cells
Source: Cancers (Basel). 2022 Apr 29;14(9):2222. doi: 10.3390/cancers14092222 (PMC9102170; doi:10.3390/cancers14092222)
Supplement: Supplementary file 1 [file cancers-14-02222-s001.zip › cancers-1674359-Supplementary Figures and Tabels_proofread.pdf]

Supplementary Materials: LPIN1 Induces Gefitinib Resistance in EGFR Inhibitor-Resistant Non-Small Cell Lung Cancer Cells

Jung Hee Cho, Yeon-Mi You, Han Koo, Dong Chul Lee, Young Il Yeom and Kyung Chan Park

Table S1. shRNA and siRNA sequences.

Name	Oligo sequence (5′-3′)
**shRNA**	
LPIN1 #1	CCGGGCAGAACTCTTCCTAATGATACTCGAG
(TRCN0000150763)	TATCATTAGGAAGAGTTCTGCTTTTTG
LPIN1 #2	CCGGCGAGAGAAAGTGGTTGACATACTCGAG
(TRCN0000153806)	TATGTCAACCACTTTCTCTCGTTTTTG
LPIN1 #3	CCGGCCAGGGTAAAGCATGAATCATCTCGAG
(TRCN0000154038)	ATGATTCATGCTTTACCCTGGTTTTTG
LPIN1 #4	CCGGCCTCAGACAGAAATGCAGTTTCTCGAG
(TRCN0000154092)	AAACTGCATTTCTGTCTGAGGTTTTTG
Non-targeting pLKO	CCGGCAACAAGATGAAGAGCACCAACTCGAG
(SCH002)	TTGGTGCTCTTCATCTTGTTGTTTTTG
**siRNA**	
LPIN1 #1	CUGACAUUUCUAAGCAAGAdTdT
LPIN1 #2	CACACAAAGGCAAACAGAUdTdT
PCKα #1	GUCACAGUACGAGAUGCdTdT
PCKα #2	CUCAAGAAGCUGAAGUGUAdTdT
PCKδ #1	CAGAAGCCGACCAUGUdTdT
PCKδ #2	CUCAUGGUACUUCCUCUGUdTdT
PCKζ #1	CUGGAUUUUCUGACAUCAAdTdT
PCKζ #2	CCUGCAGGAAGCAUAUGGAdTdT
RICTOR #1	CACCUUCUUAAUCAUCCAAdTdT
RICTOR #2	UUGGAUGAUUAAGAAGGUGdTdT

List of shRNA and siRNA sequences used for gene-silencing.

Table S2. Primer sequences.

Target gene	Sequence (5'-3')
ACTB	F: CTCTTCCAGCCTTCCTTCCT
	R: AGCACTGTGTTGGCGTACAG
LPIN1	F: CAATGGAAACCTCCAATGCT
	R: TCCACAGATTCCCCATTGAT
PRKCA	F: GTGGCAAAGGAGCAGAGAAC
	R: TGTAAGATGGGGTGCACAAA
PRKCD	F: GCCTCAACAAGCAAGGCTAC
	R: AGGTGGGGCTCATGTAGTTG
PRKCZ	F: GTTATCGATGGGATGGATGG
	R: GCACCAGCTCTTTCTTCACC
SREBF1	F: ATCGCTCCTCCATCAATGAC
	R: TTCCTCAGAACGCCAGACTT
SREBF2	F: CTGCTGTCCACAAAAGCAAA
	R: GGTCAGTGTGTCCTCCACCT
ACLY	F: TGCCGACTACATCTGCAAAG
	R: GGTTCAGCAAGGTCAGCTTC
FASN	F: CACAGGGACAACCTGGAGTT
	R: ACTCCACAGGTGGGAACAAG
RICTOR	F: TGCAACGATCTTCCTCAGTG
	R: CTCTTGCACCTCGATCATCA

List of primer sequences used in qRT-PCR. (F: forward; R: reverse).

S1

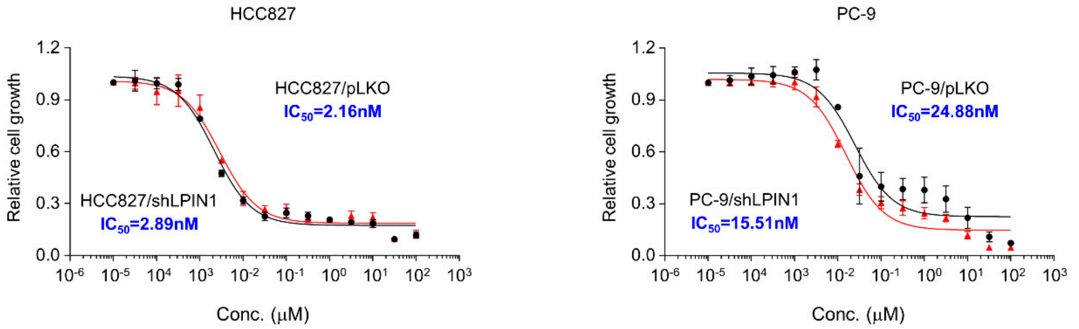

Figure S1. Determination of IC50 in HCC827 and PC-9 cells. HCC827 and PC-9 cells stably transduced with a lentiviral vector carrying shLPIN1 were treated with gefitinib or DMSO for 120 h. The gefitinib sensitivity of each cell line was determined using varying gefitinib concentrations.

S2

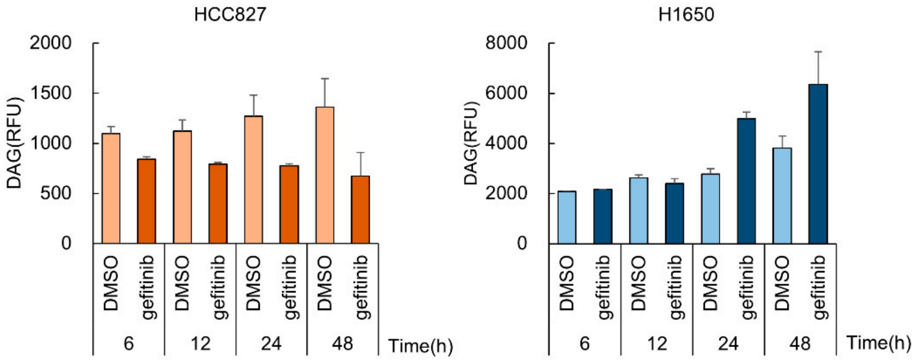

Figure S2. Determination of the amount of DAG in H1650 and HCC827 cells. H1650 and HCC827 cells were treated with gefitinib (5 μM in H1650, 0.02 μM in HCC827) or DMSO for an indicated time and then harvested to determine the amount of DAG.

S3

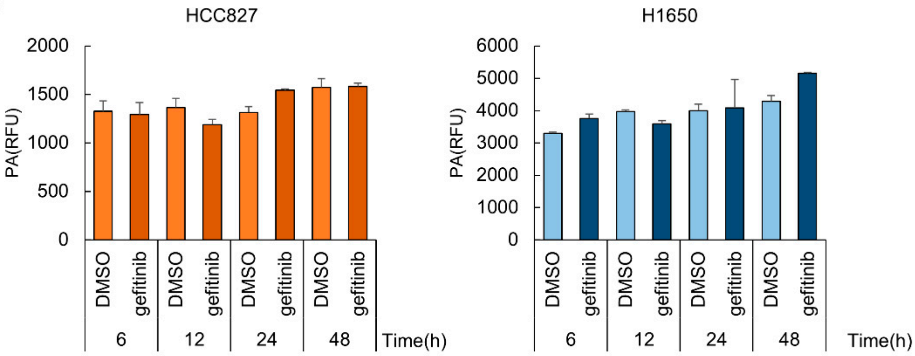

Figure S3. Determination of the amount of PA in H1650 and HCC827 cells. H1650 and HCC827 cells were treated with gefitinib (5 μM in H1650, 0.02 μM in HCC827) or DMSO for an indicated time and then harvested to determine the amount of PA.

S4

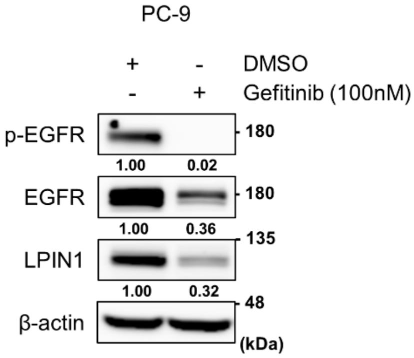

Figure S4. Determination of the protein levels of LPIN1 in PC-9 cells. PC-9 cells were treated with gefitinib (100 nM) or DMSO for 24h and then harvested to determine the protein levels of LPIN1.

S5

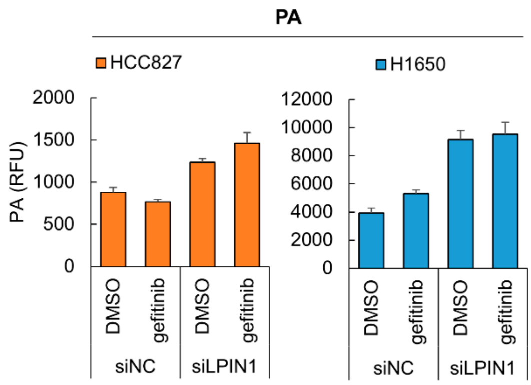

Figure S5. Determination of the amount of PA in H1650 and HCC827 cells transfected with siRNAs for LPIN1. H1650 and HCC827 cells were transfected with siRNAs for LPIN1, treated with gefitinib (5 μM in H1650, 0.02 μM in HCC827) or DMSO for 24 h, and then harvested to determine the amount of PA.

S6

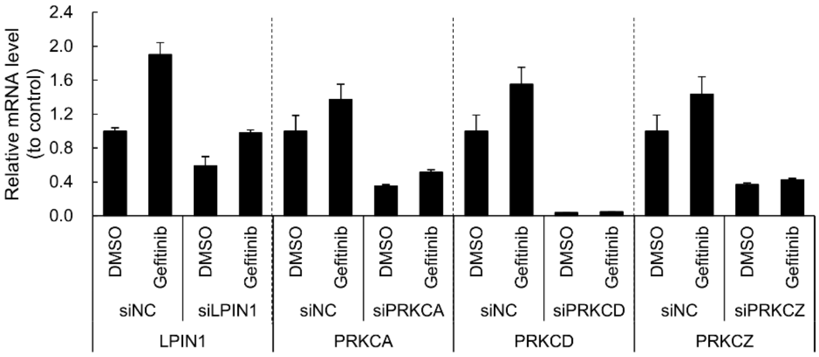

Figure S6. Determination of the levels of LPIN1, PRKCA, PRKCD, PRKCZ mRNA in H1650 cells transfected with siRNA for themselves. H1650 cells were transfected with siRNAs for LPIN1, PRKCA, PRKCD and PRKCZ and treated with gefitinib (5μM) or DMSO for 24h. Cells were harvested, and then the levels of LPIN1, PRKCA, PRKCD and PRKCZ mRNA were determined by qRT-PCR to identify knockdown efficiencies.

S7

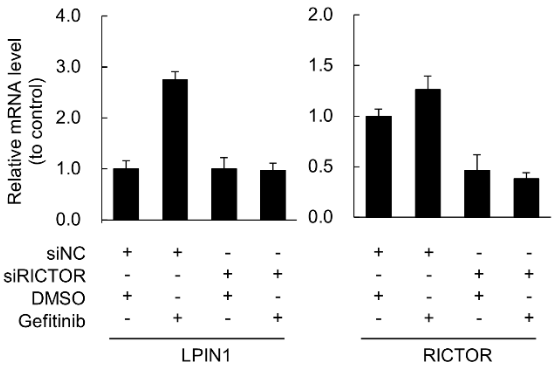

Figure S7. Determination of the levels of LPIN1and RICTOR mRNA in H1650 cells transfected with siRNAs for RICTOR. H1650 cells were transfected with siRNAs for RICTOR and treated with gefitinib (5 μM) or DMSO for 24 h. Cells were harvested, and then the levels of LPIN1 mRNA were determined by qRT-PCR.
