# Supplementary material for: LPIN1 Induces Gefitinib Resistance in EGFR Inhibitor-Resistant Non-Small Cell Lung Cancer Cells
Source: Cancers (Basel). 2022 Apr 29;14(9):2222. doi: 10.3390/cancers14092222 (PMC9102170; doi:10.3390/cancers14092222)

cancers

MDPI

Supplementary Materials: LPIN1 Induces Gefitinib Resistance in EGFR Inhibitor-Resistant Non-Small Cell Lung Cancer Cells

Jung Hee Cho, Yeon-Mi You, Han Koo, Dong Chul Lee, Young Il Yeom and Kyung Chan Park

File S1. Whole western blots.

Figure 1F

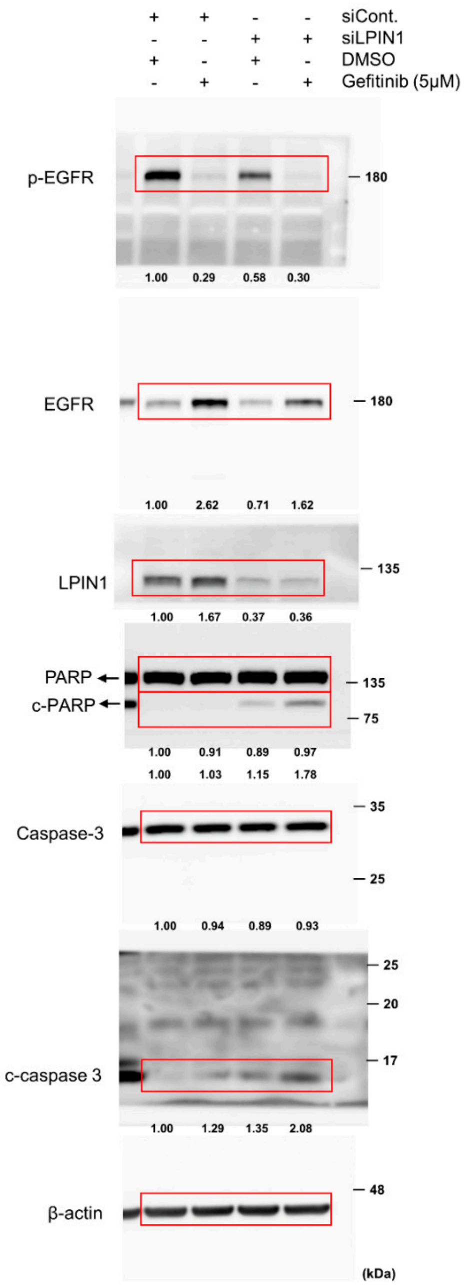

Figure 2A

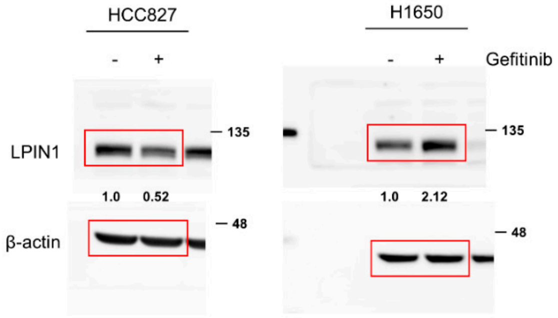

Figure S4

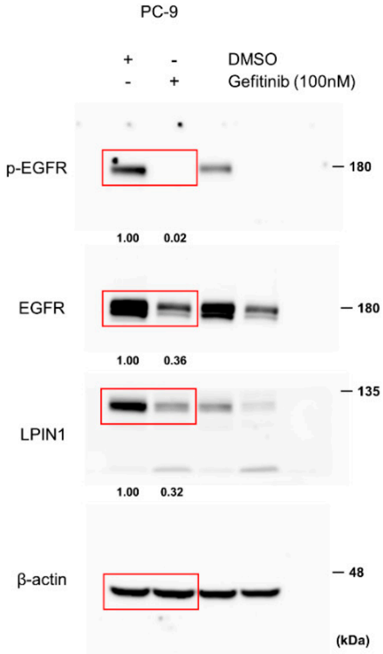

Figure 3A

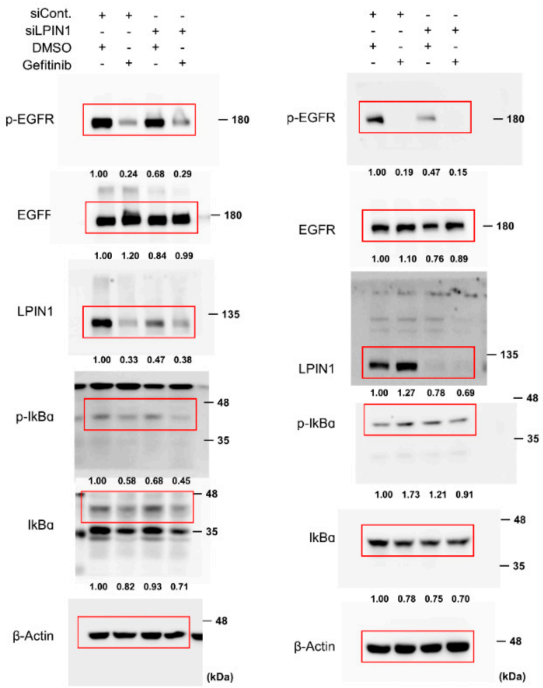

Figure 3B

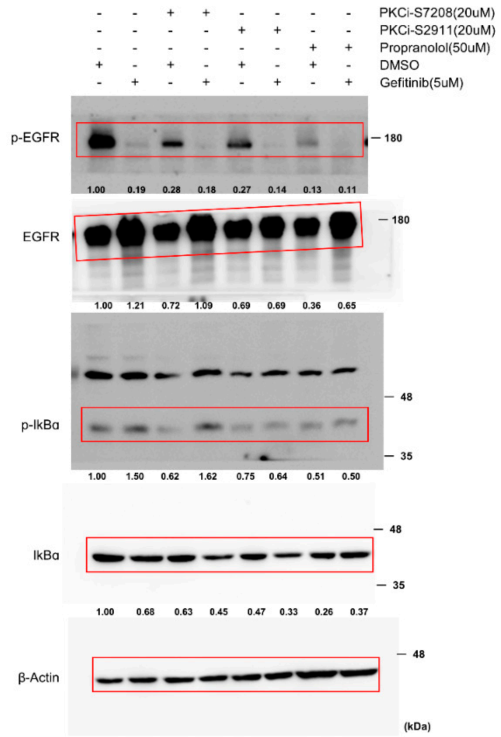

Figure 3C

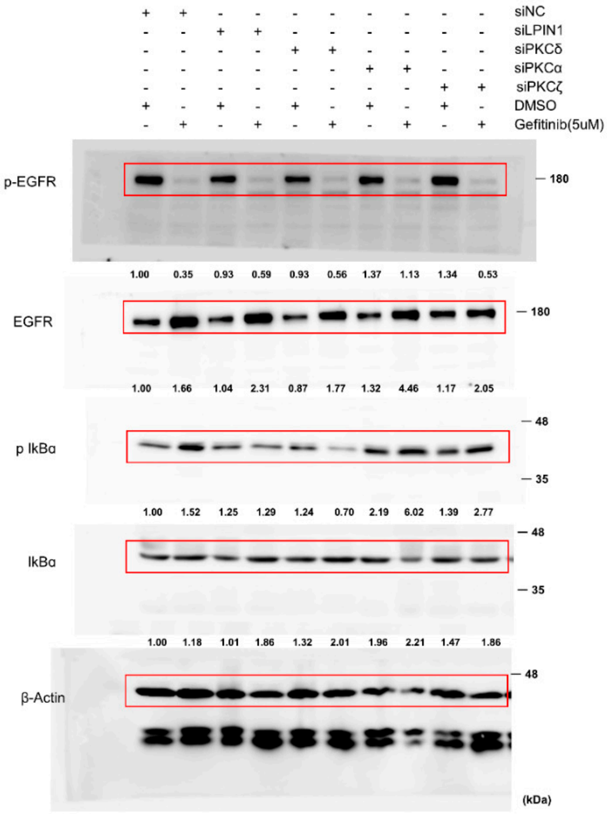

Figure 4D

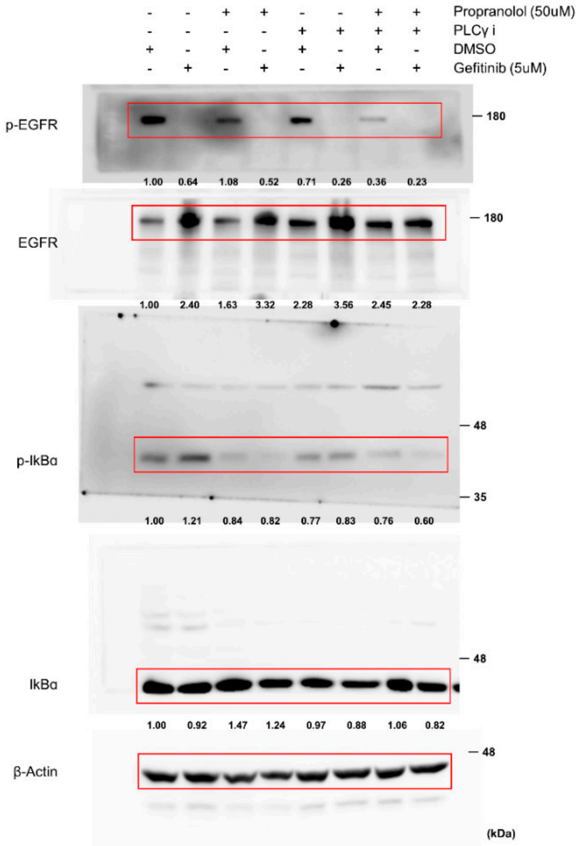

Figure 5F

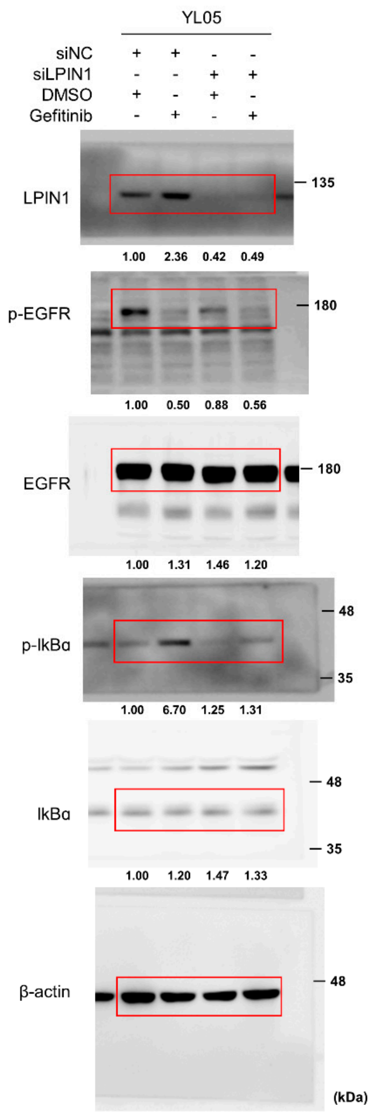

Figure 6C (upper panel)

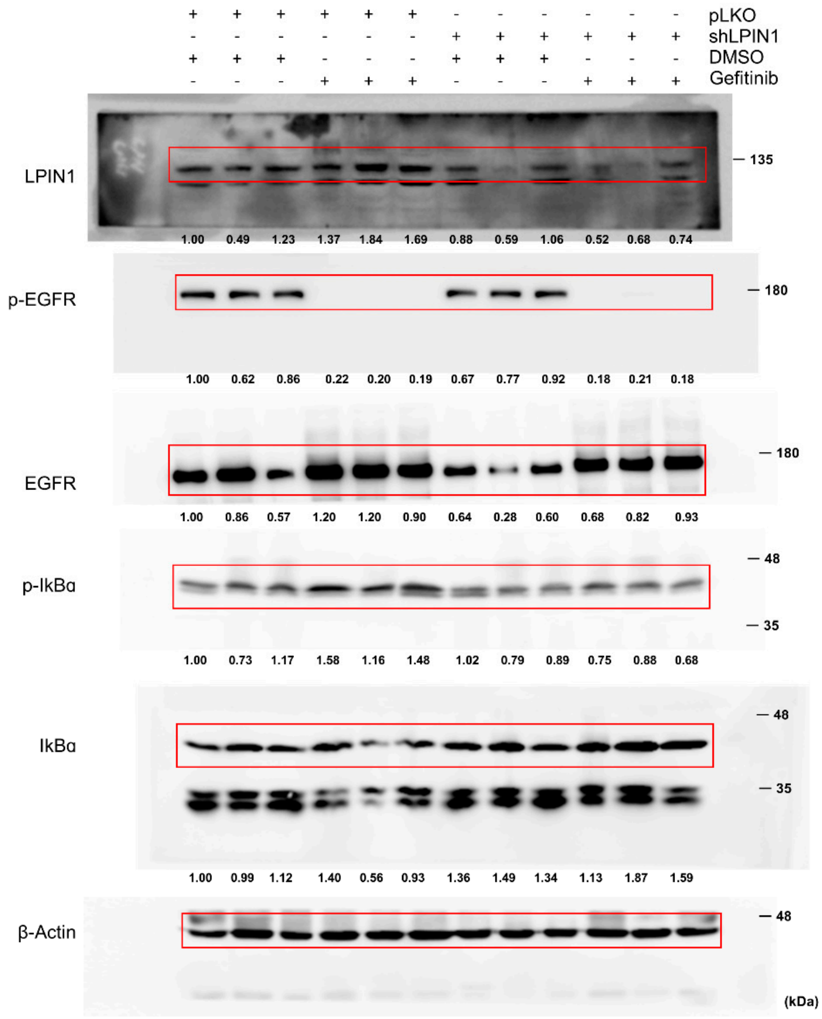

Figure 6C (lower panel)

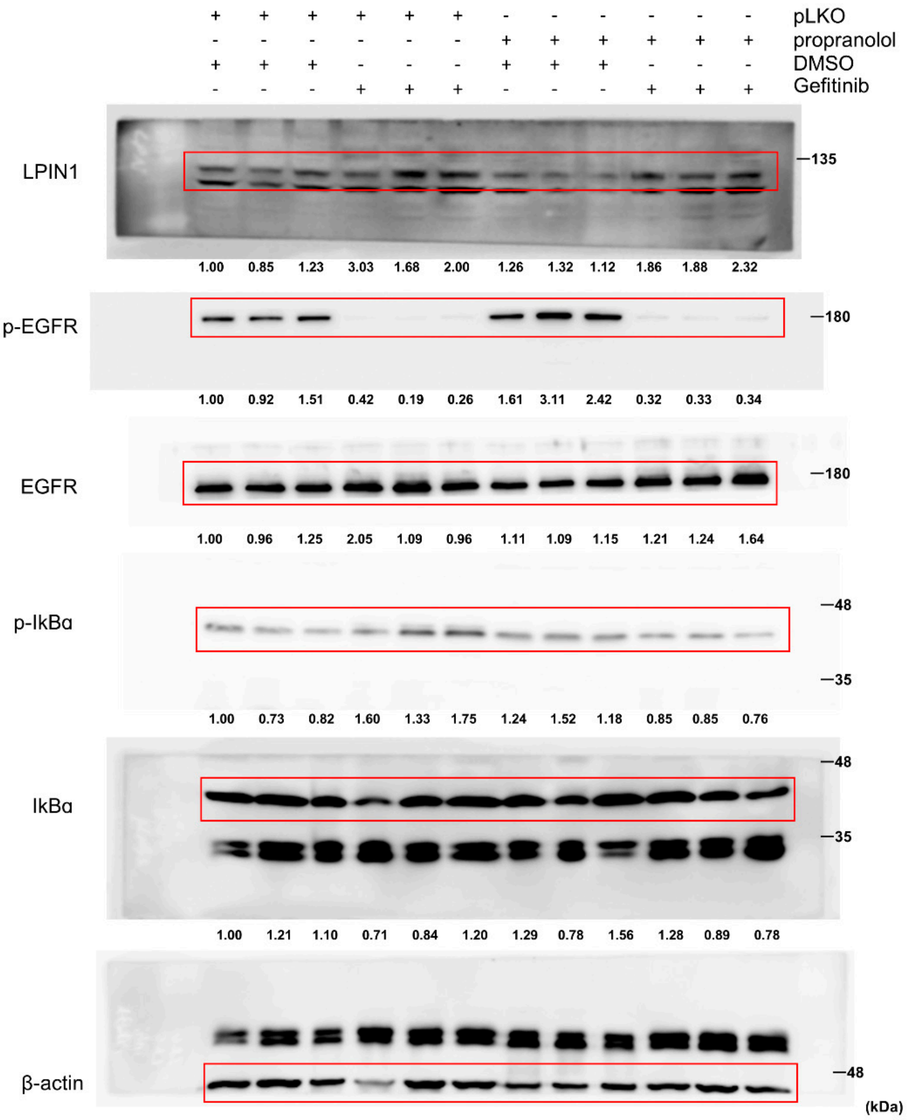

Supplement: Supplementary file 1 [file cancers-14-02222-s001.zip › File S1-Whole WB.pdf]
